# *Ericaphisvoegtlini*, a new, unusual aphid species from the USA (Hemiptera, Aphididae)

**DOI:** 10.3897/zookeys.785.28006

**Published:** 2018-09-19

**Authors:** Shalva Barjadze, Andrew S. Jensen, Mariusz Kanturski

**Affiliations:** 1 Institute of Zoology, Ilia State University, Giorgi Tsereteli 3, 0162, Tbilisi 0159, Georgia Ilia State University Tbilisi Georgia; 2 Department of Entomology, Washington State University, Pullman, Washington, USA Washington State University Pullman United States of America; 3 Department of Zoology, Faculty of Biology and Environmental Protection, University of Silesia in Katowice, Bankowa 9, 40–007 Katowice, Poland University of Silesia in Katowice Katowice Poland

**Keywords:** *
Chamaebatia
*, description, Macrosiphini, new species, taxonomy

## Abstract

*Ericaphisvoegtlini***sp. n.** living on *Chamaebatiafoliolosa* (Rosaceae) in California is described based on apterous and alate viviparous females. The new species differs from all other species of the genus *Ericaphis* Börner, 1939 in several important morphological characters including very long and rigid dorsal setae and distinctly swollen siphunculi with clearly visible polygonal reticulation.

## Introduction

The genus *Ericaphis* was established by Börner (1939) for *E.ericae* (Börner, 1933), previously described in the genus *Myzaphis* van der Goot, 1913 (Remaudière and Remaudière 1997). The genus comprises 12 species and most are Nearctic (Blackman and Eastop 2018, Favret 2018, Kanturski et al. 2018). *Ericaphis* members are characterised by well–developed median and lateral frontal tubercles, somewhat spinulous or scabrous antennae that are shorter than the body, antennal segment III usually without secondary rhinaria, first tarsal segments with 3–3–3 or 5–5–5 setae and the siphunculi characteristically S–curved, the cauda tongue or finger–shaped with few setae. Secondary rhinaria in alate viviparous females often have cilia–like fimbriation or striation (Heie 1992, Foottit and Richards 1993, Blackman 2010). The species currently placed in *Ericaphis* are a diverse group, and some species currently placed in other genera, such as *Aulacorthum* Mordvilko, 1914 and *Wahlgreniella* Hille Ris Lambers, 1949 are probably closely related to some of them.

The plant genus *Chamaebatia* Benth. (Rosaceae) is a local endemic of California, USA with two shrub species: *Chamaebatiaaustralis* (Brandegee) Abrams and *Chamaebatiafoliolosa* Benth. (Munz 1973; The Plant List 2013). Two aphid species are recorded on *C.foliolosa* – *Macrosiphumeuphorbiae* and “?*Ericaphis* sp.” (Blackman and Eastop 2018). The latter species was mentioned as “Ericaphissp. nearwakibae (California; BMNH colln, leg. D Hille Ris Lambers)” in the identification key to *Chamaebatia*–feeding aphids in Blackman and Eastop (2006, p. 251).

During work in the Aphididae collection of the Natural History Museum in London (United Kingdom) specimens of the above-mentioned *Ericaphis*–like undescribed species collected by David Voegtlin on *C.foliosa* in California, USA were found by M. Kanturski. In addition, specimens of the same species were collected by A. Jensen on the same plant and in the same area of California in 2014.

The new species, living on *Chamaebatiafoliolosa* from California, USA, is here described based on apterous and alate viviparous females sampled by David Voegtlin, D. Hille Ris Lambers, and Andrew Jensen.

## Materials and methods

The specimens were examined using light microscope Nikon Eclipse E600 with differential interference contrast (DIC) and photographed by Nikon DS–Fi camera. The measurements were done according to Ilharco and van Harten (1987) and Blackman and Eastop (2006). Measurements are given in millimetres. The following abbreviations are used:

**ANT** antennae or their lengths;

**ANT I–VI** antennal segments I, II, III, IV, V, VI or their lengths (ratios between antennal segments are simply given as e.g. ‘VI: III’);

**BASE** basal part of last antennal segment or its length;

**BD III** basal articular diameter of ANT III;

**BL** body length (from anterior border of the head to the end of cauda);

**FEMORA III** hind femora length;

**HW** greatest head width across compound eyes;

**HT I** first segment of hind tarsus;

**HT II** second segment of hind tarsus or its length;

**LS ANT III** length of longest setae of ANT III;

**PT** processus terminalis of last antennal segment or its length;

**SIPH L** siphunculi length,

**SIPH W** maximum width of swollen part of siphunculus;

**TIBIAE III** hind tibiae length;

**URS** ultimate segments of rostrum (IV + V) or their length.

Depositories of type material:

**AJ** Andrew S. Jensen Aphididae Collection (USA);

**BMNH**Natural History Museum, London (United Kingdom);

**DZUS**Hemiptera Collection of the Department of Zoology, University of Silesia in Katowice (Poland);

**ISU** Institute of Zoology at Ilia State University (Georgia);

**USNM**National Collection of Aphidomorpha, Beltsville, MD. (USA).

## Taxonomy

### Aphididae Latreille, 1802

#### *Ericaphis* Börner, 1939

##### Ericaphis voegtlini sp. n.

Taxon classificationAnimaliaHemipteraAphididae

http://zoobank.org/BBB29D9E-6287-416E-B2CD-246231AB4B57

**Figure 1.**
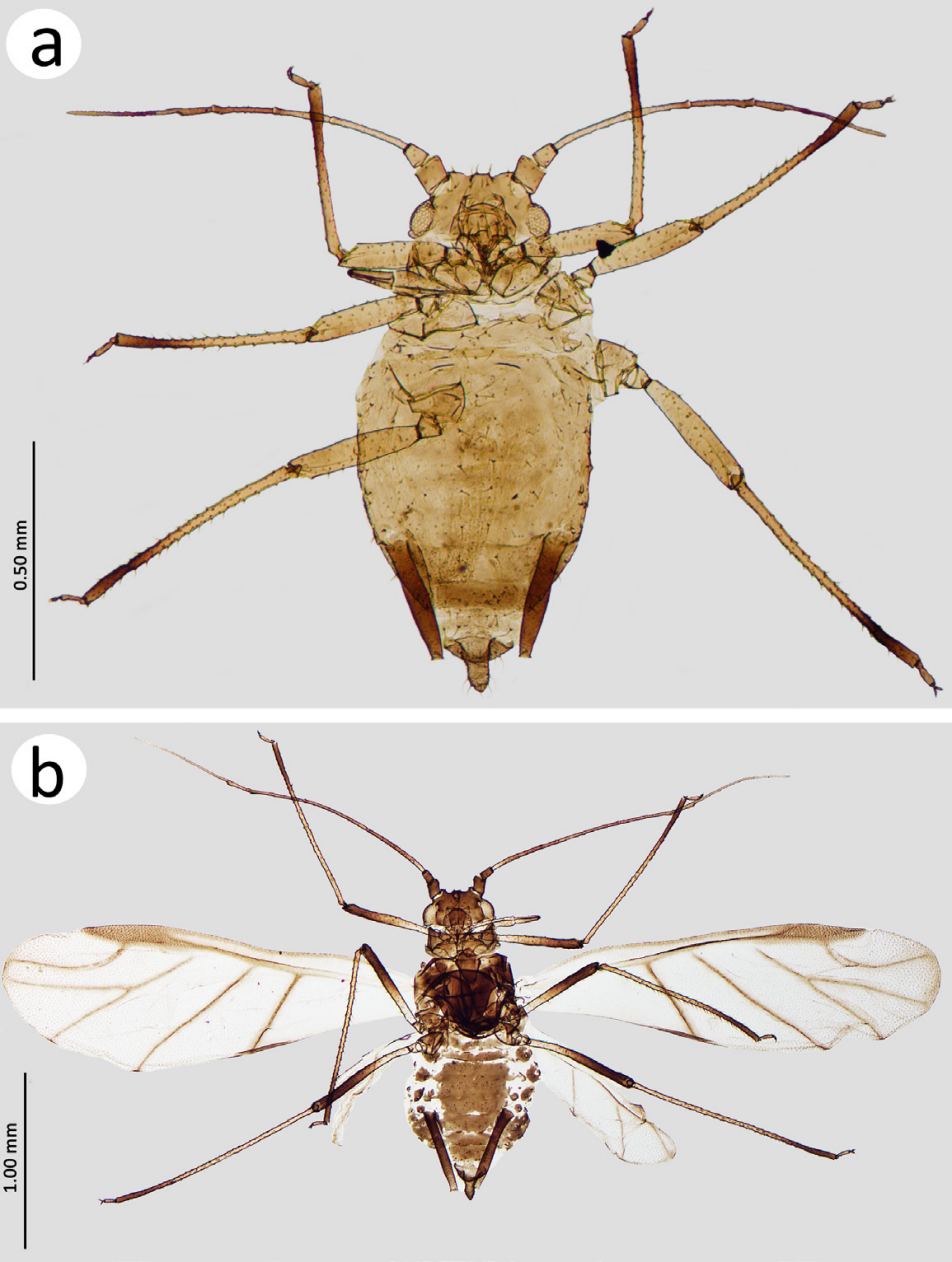
*Ericaphisvoegtlini* sp. n. General view. **a** Apterous viviparous female **b** Alate viviparous female.

**Figure 2.**
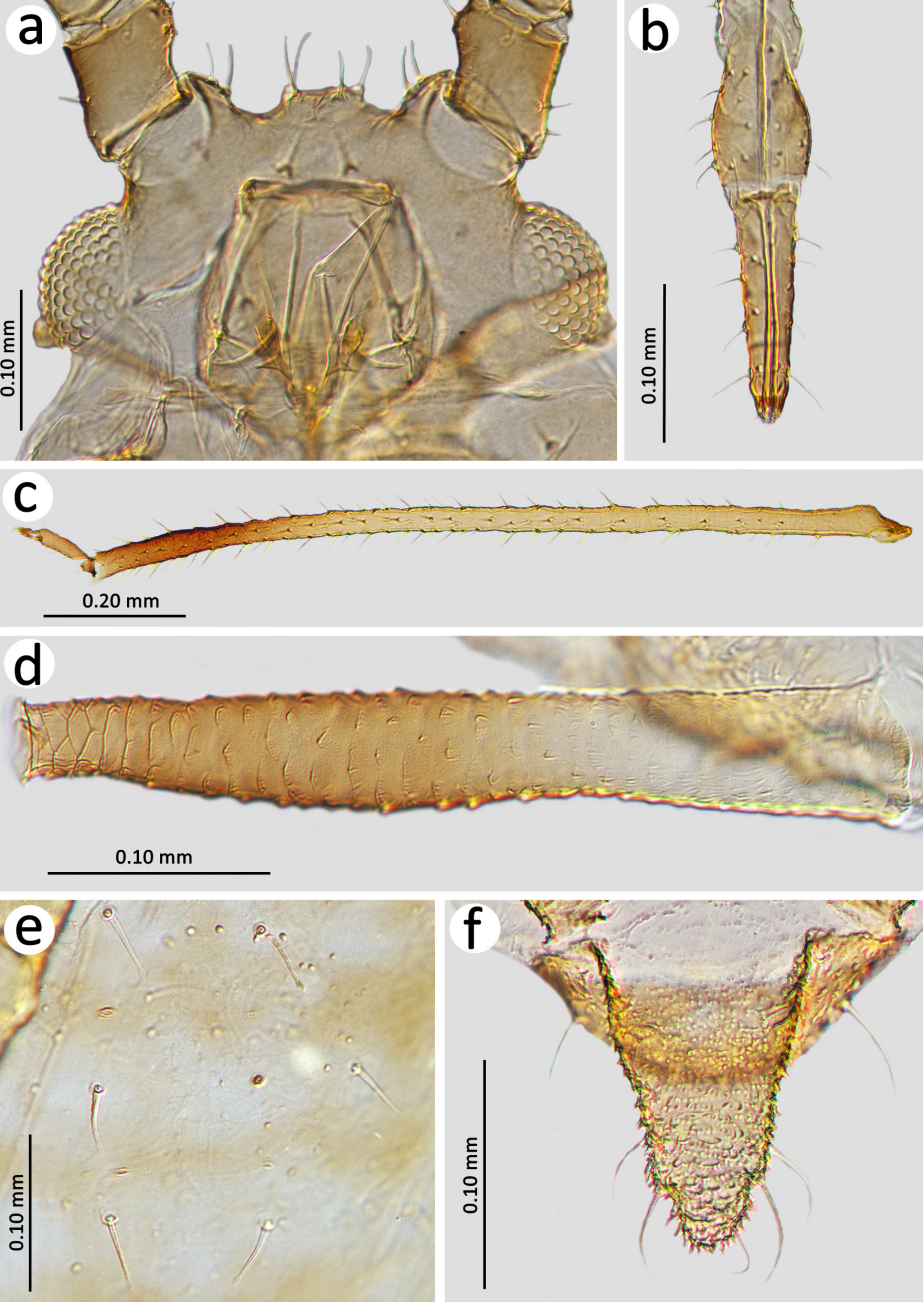
Apterous viviparous female of *Ericaphisvoegtlini* sp. n. Characters. **a** Head with long frontal and antennal tubercle setae **b** Ultimate rostral segments **c** Hind tibia **d** Siphunculus **e** Dorsal chaetotaxy on ABD II–IV **f** Cauda.

**Figure 3.**
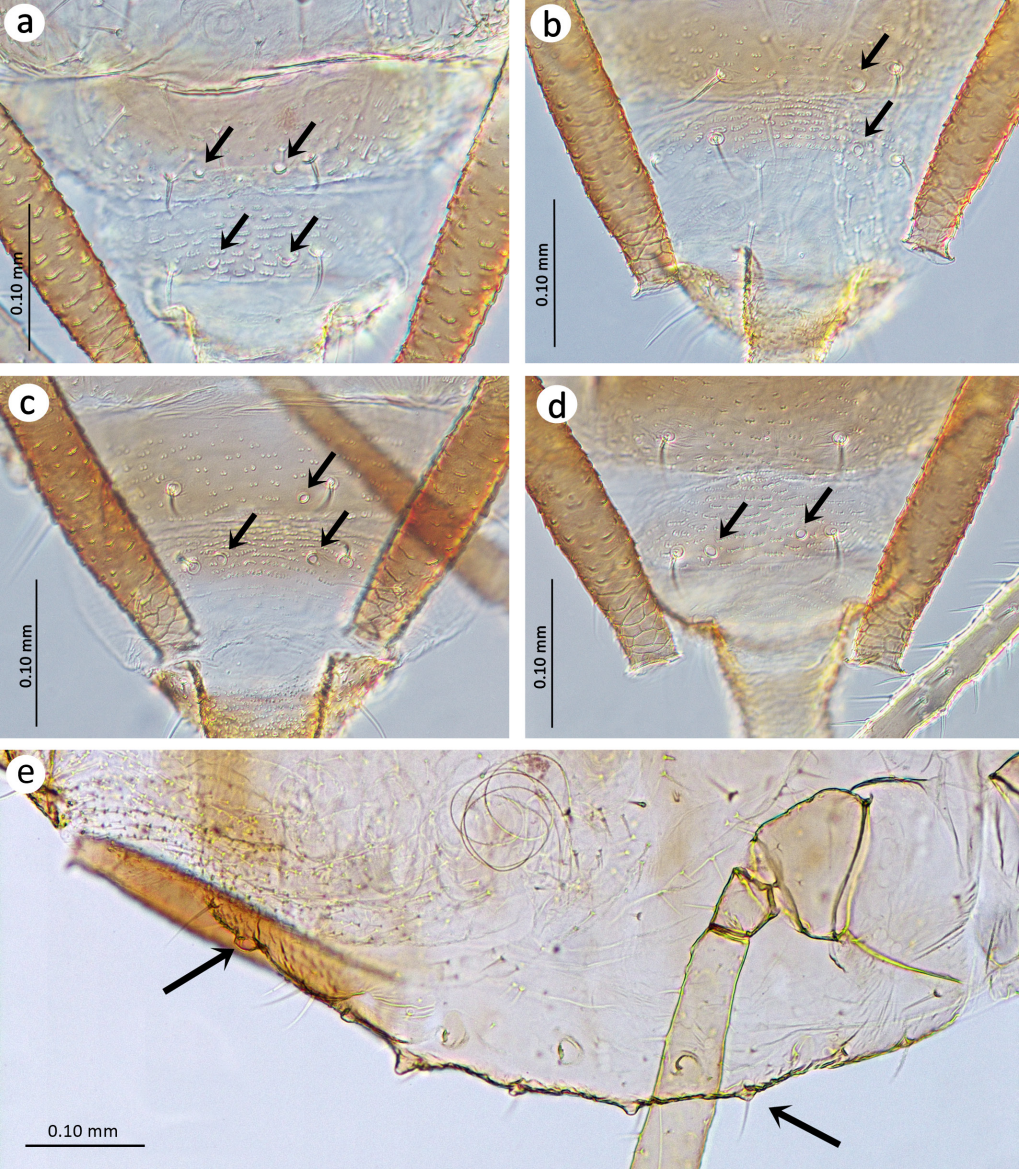
Spinal and marginal tubercles in apterous viviparous females of *Ericaphisvoegtlini* sp. n. **a** Two pairs on ABD VII and ABD VIII **b** Only one tubercle on ABD VII and ABD VIII **c** One tubercle on ABD VII and two tubercles on ABD VIII **d** Only two tubercles on ABD VIII (The location of the marginal tubercles indicated by arrows) **e** Marginal tubercles on ABD II–VII (Tubercles on ABD I and ABD VIII indicated by arrows).

**Figure 4.**
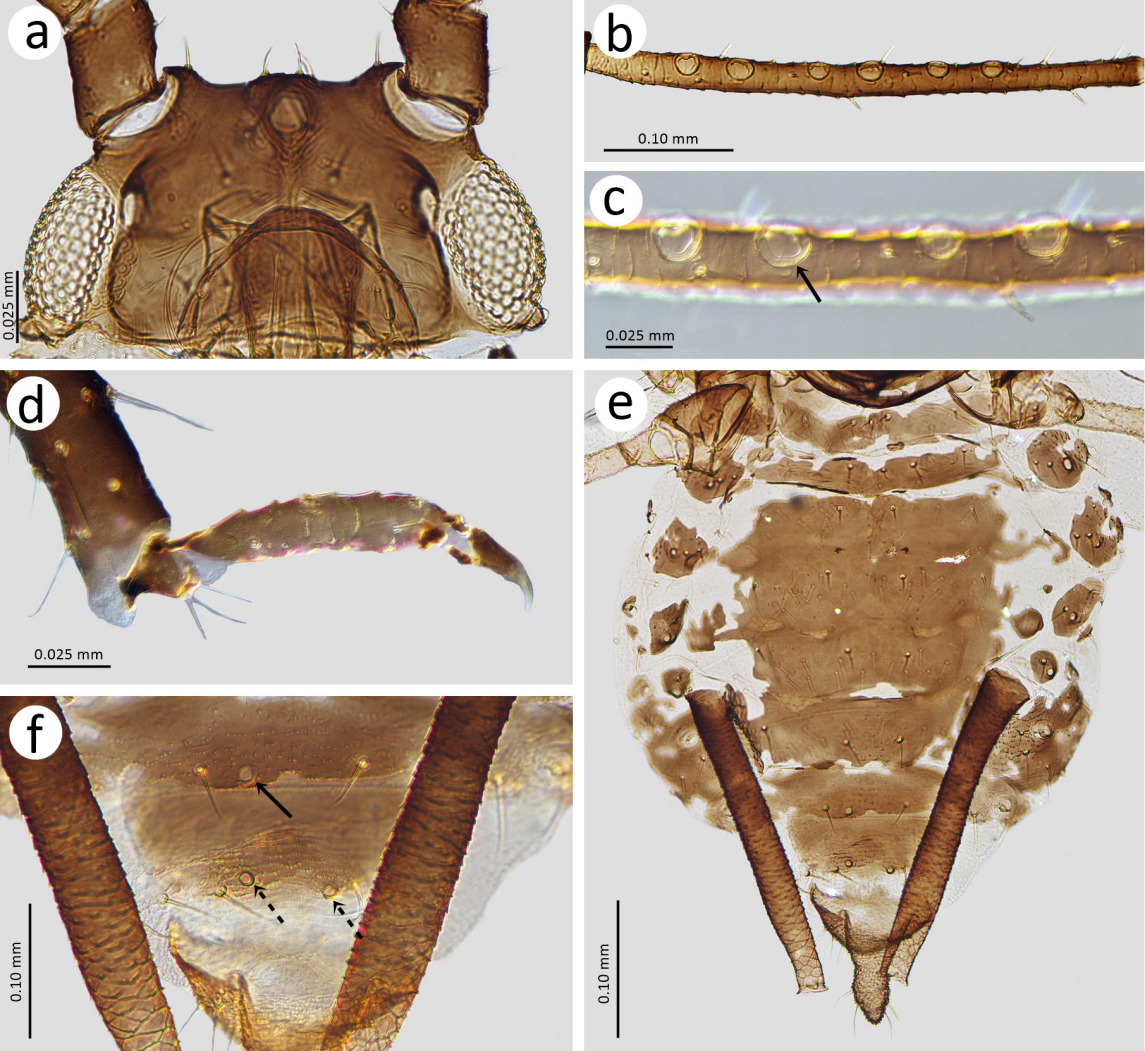
Alate viviparous female of *Ericaphisvoegtlini* sp. n. Characters. **a** Head **b**ANT III with secondary rhinaria **c** Structure of the sensoria with visible fibriation (arrow) **d** Hind tarsus **e** Abdominal sclerotisation **f** Marginal tubercles on ABD VII and ABD VIII.

Figures 1 , 2 , 3 , 4 ; Table 1

###### Diagnosis.

Apterous viviparous females differ from other *Ericaphis* by having a well–developed quadrangular median frontal tubercle, and long and rigid dorsal setae on head, thorax, and abdominal dorsum. The siphunculus is also unusually swollen and has 4–7 rows of polygonal cells in its subapical zone. The new species shares with *E.avariolosa* (David, Rajasingh & Narayanan, 1971), *E.leclanti* Remaudière, 1971, and *E.wakibae* (Hottes, 1934) some features of the siphunculus (e.g. slightly swollen with row(s) of polygonal cells in the subapical zone) but differs in the other above-mentioned characters.

**Table 1.** Measurements of apterous and alate viviparous females of *Ericaphisvoegtlini* sp. n.

**Character**	**Apterous viviparous females (n=34**)	**Alate viviparous females (n=22)**
BL	1.299–1.90	1.67–2.00
BW	0.540–0.900	0.70–0.78
HW	0.32–0.39	0.37–0.394
ANT	0.97–1.65	1.80–2.026
ANT III	0.20–0.38	0.39–0.44
ANT IV	0.14–0.30	0.35–0.4423
ANT V	0.15–0.30	0.31–0.359
ANT VI	0.34–0.50	0.57–0.69
BASE	0.085–0.110	0.12–0.146
PT	0.25–0.39	0.45–0.57
III FEMORA	0.34–0.64	0.60–0.664
III TIBIAE	0.5–1.12	1.19–1.274
HT II	0.07–0.08	0.080–0.085
Rostrum	0.35–0.49	0.44–0.492
URS	0.12–0.14	0.135–0.154
SIPH L	0.26–0.53	0.46–0.472
SIPH W (most wide part)	0.04–0.07	0.05–0.06
SIPH W (most narrow part)	0.034–0.06	0.035–0.05
Cauda L	0.125–0.22	0.15–0.175
Cauda W (at base)	0.09–0.14	0.10–0.12

###### Type material.

***Holotype***: UNITED STATES OF AMERICA, California: Calaveras Co., Board’s Crossing, Stanislaus N. F. (38°18'13"N; 120°14'54"W, 1180 m a.s.l.), 15 April 2014, on *Chamaebatiafoliolosa*, A. Jensen leg., 1 apterous viviparous female marked as holotype (“H”) and circle on the slide, AJ7029, USNM. ***Paratypes***: the same data as the holotype, 2 apterous viviparous females, AJ7029, AJ; 3 apterous viviparous females, AJ7032, AJ; 4 apterous viviparous females, AJ7030, AJ; Sheep Ranch Rd. near Avery (38°12'02"N; 120°23'52"W, 1086 m a.s.l.), 15 April 2014, on *Ch.foliolosa*, A. Jensen leg., 2 apterous viviparous females, AJ7019; Placer Co., 3 mi. S.W. Dutch Flat HWY 80 (39°11'14"N; 120°50'47"W, 972 m a.s.l.), 22 April 1978, on *Ch.foliolosa*, D. Hille Ris Lambers no 33 leg., 1 apterous viviparous female, BM 1984–340, BMNH; near Dutch Flat (39°11'14"N; 120°50'47"W, 972 m a.s.l.), 22 April 1978, on *Ch.foliolosa*, D. Hille Ris Lambers no 31 leg., 3 apterous viviparous females, BM 1984–340 (1 – present marking), DZUS; 3 mi. S.W. Dutch Flat HWY 80 (39°11'14"N; 120°50'47"W, 972 m a.s.l.), 23 May 1978, on *Ch.foliolosa*, D. Hille Ris Lambers (culture) leg., 2, 1, 2, 2, 2, 2, 2, 2, 2, 2 alate viviparous females (11 slides with the same data and number), BM 1984–340, BMNH; 3 alate viviparous females BM 1984-340, DZUS; El Dorado Co., Sand Mtn. Blodgett (38°54'22"N; 120°39'30"W, 1349 m a.s.l.), 21 August 1974, on *Ch.foliolosa*, D. Voegtlin leg., 2, 2, 2, 2, 2, 2, 2, 2, 2 apterous viviparous females (9 slides with the same data and number), BM 1984–340, BMNH; 2 apterous viviparous females, BM 1984–340, ISU; Sand Mtn. Blodgett (38°54'22"N; 120°39'30"W, 1349 m a.s.l.), 21 August 1974, on *Ch.foliolosa*, D. Voegtlin leg., 2 alate viviparous females, BM 1984–340, ISU; Sand Mtn. Blodgett (38°54'22"N; 120°39'30"W, 1349 m a.s.l.), 21 August 1974, on *Ch.foliolosa*, D. Voegtlin leg.

###### Description.

*Apterous viviparous female* (n = 34). Colour in life: dark green. On slide: body in general sclerotised, pale yellow to yellow. ANT yellow with brown distal part of ANT IV and whole ANT V–VI. Tibiae yellow with brown to dark brown distal parts (but the very apex of tibiae lighter). Tarsi light brown to brown. SIPH pale to yellow basally and brown to dark brown distally (Figure 1a).

Slide–mounted specimens: HW 0.23–0.36 × ANT. Head chaetotaxy: head with three dorsal pairs of setae; median tubercle with 4–5, ANT tubercles each with 3–5 long, rigid, thick setae with blunt or narrow capitate apices, 0.017–0.052 mm long. Frontal setae 0.037–0.050 mm long (Figure 2a). ANT 0.70–1.04 × BL. ANT III without secondary rhinaria, ANT IV slightly shorter or slightly longer than ANT V. ANT V with ciliated primary rhinarium at the distal part. PT 3.00–3.54 × BASE. Other antennal ratios: VI:III 1.31–1.87, V:III 0.67–0.78, IV:III 0.66–0.81, PT:III 1.02–1.45, PT:IV 1.30–1.93, PT:V 1.30–1.93. ANT chaetotaxy: ANT bearing very short and rigid setae with blunt apices. ANT III setae shorter than the width of the segment, 0.007–0.020 mm long, LS III 0.41–0.90 × BD III. ANT I with 7–10, ANT II with 4–5, ANT III with 11–17, ANT IV with 7–16, ANT V with 7–12 setae. ANT VI with 3–5 basal, 3 apical and 5–6 setae on the PT. Rostrum reaching from metasternum to ABD I. URS 0.36–0.60 × ANT III, 0.35–0.47 × PT, 1.20–1.50 × BASE and 1.66–1.87 × HT II with 11–19 fine and pointed accessory setae (Figure 2b). Mesosternal furca fused, wide, Y–shaped. III FEMORA bearing short, thick, rigid setae with ragged or pointed apices, 0.010–0.035 mm long. III TIBIAE bearing thick, rigid setae with ragged or flat apices, shorter than the width of tibiae, 0.007–0.041 mm long (Figure 2c). HT I with 3–3–3 ventral setae, HT II 0.21–0.35 × ANT III, 0.20–0.27 × PT and 0.70–0.82 × BASE. SIPH tubular, slightly curved, swollen from about mid–length with distinct zone of subapical reticulation formed from 4–7 rows (Figure 2d). The reticulated zone 0.07–0.16 × SIPH. SIPH 2.08–2.76 × cauda, 0.18–0.32 × BL, and 1.24–1.60 × ANT III. Abdomen sclerotised with long and thick setae in spinal, pleural and marginal positions. Dorsal setae with capitate apices, 0.015–0.047 mm long on ABD I–V and 0.040–0.065 mm long on ABD VI–VIII (Figure 2e). ABD VII with 0–2 and ABD VIII with 1–2 spinal tubercles (Figures 3a–d). Marginal tubercles on ABD II–VII, sometimes poorly–visible (Figure 3e). ABD VIII usually with 4–5 setae. Genital plate with two anterior setae which are longer than the others, 6–12 setae in the middle and 9–12 posterior setae. Cauda 1.30–2.00 × its width at base and 0.09–0.12 × BL, with 5–7 long and fine setae (Figure 2f).

*Alate viviparous female* (n=22). Colour in life: unknown. On slide: head and ANT light brown to brown with basal part of Ant III and PT lighter. Pronotum light brown, rest of thorax brown. Wings hyaline with light brown veins (cubital veins slightly darker). Femora brown with pale proximal part and dark distal part. Tibiae yellow to light brown with brown to dark distal part. Abdomen with brown sclerotisation, SIPH brown with lighter apical part, cauda brown (Figure 1b).

Slide–mounted specimens: HW 0.18–0.21 × ANT. Head chaetotaxy: head with four dorsal pairs of long, rigid, thick setae with capitate apices, 0.022–0.050 mm long. Frons with four setae, ANT tubercles with 2–4 setae (Figure 4a). ANT 1.01–1.12 × BL. ANT III with 6–9 secondary rhinaria (Figures 4b, c), ANT IV longer than ANT V without secondary rhinaria. ANT V with primary rhinarium with ciliated rim. PT 3.60–4.66 × BASE. Other antennal ratios: VI:III 1.43–1.74, V:III 0.72–0.87, IV:III 0.81–1.02, PT:III 1.12–1.43, PT:IV 1.25–1.53, PT:V 1.27–1.75. ANT chaetotaxy: ANT with short and rigid setae with blunt apices. ANT III setae shorter than the width of the segment, 0.015–0.017 mm. LS III 0.68–0.87 × BD III. ANT I with 7–11, ANT II with 4–5, ANT III with 14–20, ANT IV with 12–16, ANT V with 9–12 setae. ANT VI with 3–4 basal, 3–4 apical and 5–8 additional setae on the PT. Rostrum reaching mesosternum. URS 0.31–0.38 × ANT III, 0.25–0.30 × PT, 1.03–1.16 × BASE and 1.58–1.88 × HT II with 15–18 fine and pointed accessory setae. III FEMORA bearing thick, rigid setae with pointed or blunt apices, 0.015–0.027 mm long. III TIBIAE bearing long, slightly rigid, pointed or blunt setae, shorter or longer than the width of tibiae, 0.012–0.045 mm long. HT II (Figure 4d) 0.18–0.21 × ANT III, 0.14–0.18 × PT and 0.56–0.70 × BASE. SIPH 2.67–3.06 × cauda and 0.23–0.27 × BL. Abdomen with two crossbars on ABD I and II and a large spino–pleural sclerotic patch on ABD III–VIII and pleuro–marginal sclerites on ABD V–VII (Figure 4e). Dorsal setae long, thick, and rigid with pointed or slightly blunt apices, 0.017–0.049 mm long on ABD I–VI and 0.037–0.067 mm long on ABD VII–VIII. ABD VII and VIII with spinal tubercles (Figure 4f). ABD VIII with 4–5 setae. Subgenital plate with 19–23 setae. Cauda length 1.41–1.51 × width at base, with 5–6 setae.

###### Etymology.

The authors have the pleasure in naming the new species to honour Dr. David Voegtlin, an aphid specialist from the Illinois Natural History Survey (University of Illinois, Urbana–Champaign, USA), who was also the first collector.

###### Biology and distribution.

The new aphid species is associated with *Chamaebatiafoliolosa* Benth. (Rosaceae). Its sexual morphs and life history are unknown, but probably it is monoecious holocyclic. The aphid species is presumably endemic to California, as is its host plant.

## Discussion

Appropriate generic placement of this species was challenging due to its unusual features, including its prominent median frontal tubercle, robust dorsal setae, and swollen reticulated siphunculi. It was tempting to consider this aphid the first of a new genus. In the end we opted for placement in *Ericaphis* for a few reasons. First, *E.voegtlini* shares important features with most *Ericaphis*, including the presence of a median frontal tubercle (albeit more extreme), typically two pairs of lateral setae on the cauda (plus one dorso–apical seta), dorsal pigmented abdominal patch in the alate vivipara, no secondary rhinaria on antennal segment III in the apterous vivipara, and relatively few and large secondary rhinaria on antennal segment III in the alate vivipara. Second, there are western North American species scattered among at least three genera (*Aulacorthum*, *Ericaphis*, and *Wahlgreniella*) that share many features and that may be closely related. Ideally, the generic classification of these aphids should be done as a more comprehensive study, using all the similar and related species currently placed in these, and possibly other, genera. Third, the aphids of western North America are still in need of basic discovery work in the field. The second author spends hundreds of hours each year collecting aphids and making field observations, and has discovered, and continues to discover, many new species and host plant relationships (see http://aphidtrek.org/), including isolated samples and observations of aphids related to the above–mentioned genera. Comprehensive analysis of the relevant generic classification, therefore, is best delayed until more of the currently undescribed and undiscovered aphid species are documented and described. In the meantime, *E.voegtlini* is a distinctive, easily recognised species that feeds on an unusual plant with a very limited distribution, and interim placement of it in *Ericaphis* is a practical choice. We hope that by publishing its description we will draw attention to the possible undiscovered diversity of this aphid group in North America.

## Supplementary Material

XML Treatment for Ericaphis voegtlini
